# Deep eutectic solvent (DES) pretreatment and lignin regeneration for the development of a bamboo leaf-based bioplastic

**DOI:** 10.3389/fbioe.2024.1484585

**Published:** 2024-10-09

**Authors:** Chao Liu, Hongfei Liu, Huijie Wang, Zhaochuan Yu, Ming Yan, Xuelian Zhou, Renai Li

**Affiliations:** ^1^ International Innovation Center for Forest Chemicals and Materials and Jiangsu Co-Innovation Center of Efficient Processing and Utilization of Forest Resources, Nanjing Forestry University, Nanjing, China; ^2^ Institute of Chemical Industry of Forest Products, Chinese Academy of Forestry, Jiangsu Province Key Laboratory of Biomass Energy and Materials, Nanjing, China

**Keywords:** bamboo leaf, deep eutectic solvent, lignocellulose, bioplastic, biodegradable

## Abstract

The excessive utilization of petroleum-based plastic products has led to a pervasive environmental and human health threat. In response, the adoption of bioplastics derived from biomass has emerged as the foremost alternative to conventional plastics, owing to their inherent biodegradability and sustainability. The present study demonstrates the preparation of a biodegradable and cost-effective lignocellulosic bioplastic by utilizing dissolving bamboo leaf powder with deep eutectic solvents (DES) and regenerating lignin *in situ*. The DES was synthesized through a one-step heating and stirring method using choline chloride (ChCl) and anhydrous oxalic acid. The crystallinity of the bioplastics is enhanced by DES pretreatment, thereby improving the internal structural order of the material. Moreover, lignin regeneration reduces the pore size within the bioplastics and contributes to a more compact internal structure. The prepared lignocellulosic bioplastics exhibit remarkable mechanical strength, with a tensile strength of 113 MPa. Additionally, they demonstrate good water stability, as evidenced by a contact angle of 55.52°. Moreover, these bioplastics possess an exceptional biodegradability with a degradation rate exceeding 98% after 60 days. This study presents an innovative approach for the high-value utilization of bamboo leaf resources.

## 1 Introduction

Petroleum-based plastics have been extensively utilized due to their robustness, cost-effectiveness, and impermeability (Li L. et al., 2020; Zimmermann et al., 2020), thus significantly contributing to the convenience of individuals’ daily lives. However, the escalating consumption of petrochemical resources has led to a pressing issue in today’s society - energy and resource scarcity (Cole et al., 2013). Simultaneously, the accumulation of petroleum-based plastic waste has given rise to severe environmental concerns (Hahladakis et al., 2018). In particular, microplastics derived from petroleum-based plastics not only contaminate the soil but also infiltrate the food chain, posing a threat to human health (Koelmans et al., 2022). Therefore, the exploration and advancement of renewable and eco-friendly bioplastics has emerged as a prominent area of contemporary research. In recent years, biodegradable plastics have garnered significant attention as a novel class of environmentally-friendly materials due to their inherent characteristics encompassing degradability, renewability, and sustainability (Lambert and Wagner, 2017). Bioplastics are typically derived from biomass sources, such as corn starch, cellulose-based materials, wood, straw, and soybean protein (Ci et al., 2024a; Khan et al., 2017; Liang et al., 2022; Liu et al., 2020). These raw materials undergo chemical modification, blending, plasticization, and other techniques to prepare bioplastics (Dong T. et al., 2023; Luan et al., 2019; Qin et al., 2022). Consequently, the efficient utilization of bioplastics can contribute to a reduced reliance on fossil resources.

The cellulose, being the most abundant natural polymer on our planet, utilizes β-1, 4-glucoside bond to connect two dehydrated glucose units (cellobiose) as a recurring element (Edgar et al., 2001; Schilling et al., 2010). This remarkable feature renders it an epitome of environmental friendliness, renewability, cost-effectiveness and exceptional biocompatibility (Liu et al., 2023). However, the presence of a substantial number of hydrogen bonds between cellulose molecular chains and its highly crystalline aggregate state structure poses significant challenges in terms of dissolution and melting processes, thereby impeding the direct utilization of cellulose for biomass material preparation. Consequently, cellulose derivatives such as cellulose esters or cellulose ethers have been predominantly employed for the production of bioplastics in order to overcome their inherent processing challenges (Aziz et al., 2022; Tu et al., 2021). In recent years, the advancement of cellulose dissolution systems, such as amine oxide systems, alkali/urea solutions, ionic liquids, and deep eutectic solvents (DES), has opened up possibilities for the direct utilization of plant fibers in the synthesis of bioplastics (Budtova and Navard, 2016; Chen et al., 2019; Lindman et al., 2010; Wu C. et al., 2023; Zhu et al., 2006). For instance, the research conducted by Peelman et al. demonstrated the potential of cellulose-based films as a viable alternative for diverse food packaging applications (Peelman et al., 2013).

Bamboo resources represent not only natural biomass sources, but also high-quality cellulose reservoirs (Zhao et al., 2024). Particularly, owing to its distinctive physical properties as a natural material, bamboo exhibits extensive applicability in various domains such as construction, furniture, and transportation (Li Z. et al., 2020; Yuan et al., 2017; Zhao et al., 2023). Due to its exceptional quality compared to hardwood and other grasses, bamboo fiber is widely regarded as an ideal raw material for paper production (Cui et al., 2022; Dong D. et al., 2023). Bamboo resources are abundant worldwide, particularly in China. In addition to bamboo trunks, bamboo leaves also offer a rich source of raw material. Following decomposition, the majority of these leaves are transformed into premium organic fertilizer, thereby providing valuable nutrients for agricultural purposes (Cheng et al., 2023). Simultaneously, certain researchers are dedicated to extracting specific components from bamboo leaves for application in the pharmaceutical industry and thus realizing partial resource utilization of fallen bamboo forest foliage (Wu et al., 2012; Wu Y. et al., 2023). Furthermore, bamboo leaves possess a significant abundance of lignocellulosic constituents (Cheng et al., 2023), rendering them an optimal candidate for comprehensive biomass utilization. However, the current utilization of residual bamboo leaves after treatment remains inadequate, with a majority being incinerated as fuel or directly discarded and buried, resulting in significant resource wastage. Therefore, it is imperative to explore comprehensive approaches for the high-value utilization of bamboo leaf residues to maximize the efficient use of this valuable resource.

Cellulose-based bioplastics have gained widespread adoption in various industrial sectors and daily life due to their numerous advantages. However, the conventional manufacturing process of cellulose-reinforced composite bioplastics and cellulose films involves lignin removal from the material, resulting in the disposal of extracted lignin as manufacturing waste. This separation step is laborious and leads to wastage of the lignocellulosic component of biomass. Moreover, lignin itself can serve as a cohesive agent within the cellular matrix of plant fibers (Liu et al., 2020). The present study involves the pulverization of bamboo leaves into powder and their dissolution in deep eutectic solvents (DES). Subsequently, lignocellulosic bioplastics were synthesized through an in-situ lignin regeneration method using the abundant and cost-effective bamboo leaf powder. Furthermore, the influence of DES on the properties of the prepared bioplastics was thoroughly investigated. This research will attempt to establish a strong practical solution for efficient utilization of bamboo leaf resources and development of novel environmentally friendly plastic materials.

## 2 Materials and methods

### 2.1 Materials and chemicals

Bamboo leaves were provided from Anji County, Huzhou City, Zhejiang Province, China. Choline chloride (C_5_H_14_ClNO, 98%), sulfuric acid (H_2_SO_4_, 98%), oxalic acid (C_2_H_2_O_4_, 98%), and 1-ethyl-3-methylimidazole acetate (C_8_H_14_N_2_O_2_, 97%) were all of analytical grades and procured from Shanghai Maclin Biochemical Technology Co. Ltd., China. All chemicals were used as received without any purification.

### 2.2 Pretreatment of bamboo leaves by using DES

**FIGURE 1:**
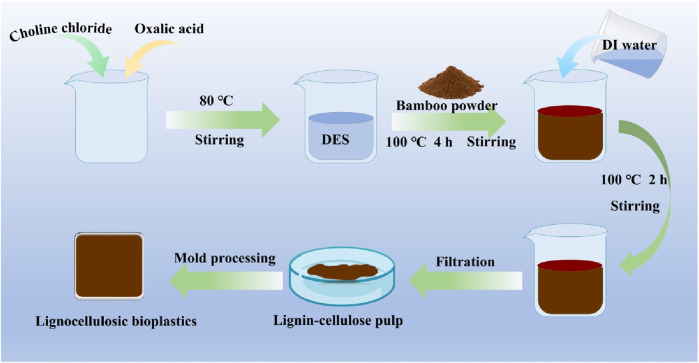
A flowchart of experiments for the preparation of lignocellulose bioplastic.

First, bamboo leaves were washed by using distilled water to remove impurities on the surface and dried at room temperature. The dried bamboo leaves were pulverized into fine particles using a grinder (DF-25, Wenling Linda Machinery Co., LTD., China), and subsequently sifted to obtain bamboo powders with a particle size ranging from 40 mesh to 60 mesh using a standard sieve. Subsequently, the uniform and clear DES was formed by mixing choline chloride (ChCl) and oxalic acid (OA) at a molar of 1:1 with a mechanical agitation under 80 °C. After the above procedure, 1 g of bamboo powder was immersed into 10 g DES and heated at 100 °C with constant mechanical stirring for 4 h, 6 h and 8 h. Then, 50 mL distilled water was added to the above mixture at 100 °C with continuous mechanical stirring (Figure 1).

### 2.3 Preparation of lignocellulose bioplastic

The lignocellulose slurry was collected by filtering and washing the above mixture. Then, the lignocellulose slurry was placed on the hydrophobic substrate mold and dried at room temperature before demolding. A lignocellulose bioplastic with a length of ∼20 mm and a thickness of ∼2 mm was obtained (Figure 1). The obtained lignocellulose bioplastic was immersed in a solution of 1-ethyl 3-methylimidazole acetate, followed by treatment at a temperature of 90 °C for a duration of 1 h. Subsequently, the lignocellulose bioplastic was immersed in ethyl alcohol and dried at room temperature.

### 2.4 Testing and characterization

#### 2.4.1 Chemical structure and micromorphology characterization

The chemical constituents containing cellulose, hemicellulose, lignin, ash, and benzene-alcohol extractive of all samples were quantified according to the standards reported by the National Renewable Energy Laboratory (NREL) (Liu C. et al., 2019; Yan et al., 2020). The morphology and structure of lignocellulose bioplastics were observed by employing a JEOL-JEM 7600F scanning electron microscope (SEM). The X-ray diffraction (XRD) of lignocellulose bioplastic samples was determined by using an X-ray refractometer (Ultima IV, Japan) with CuKa radiation (40 kV and 30 mA) in the range from 5° to 60°. The crystallinity degree (C*r*) of lignocellulose bioplastic samples was calculated in the equation based on the Segal method (Cerro et al., 2020; Nelson and O'Connor, 1964). The binding mode of C, H, O, and N atoms in the sample was analyzed by using X-ray photoelectron spectroscopy (XPS) analysis. The rheological properties of cellulose-lignin slurry with different solid contents (5%, 10%, and 15% wt%) were evaluated using a rheometer (MARS60, Germany). The FT-IR spectra were recorded in the range of 500–4,000 cm^−1^ using a VERTEX 80V FT-IR spectrometer. Additionally, sample zeta potential was determined utilizing a nano-laser particle size analyzer (Malvern nano ZSE, Malvern, United Kingdom).

#### 2.4.2 Thermal and mechanical stability, hydrophobicity, and biodegradability tests

Thermogravimetric analyses (TGA) of lignocellulose bioplastic samples were carried out by using a thermogravimetric analyzer (NETZSCH, TGA 209F1, Germany) under a 25 mL/min nitrogen flow from 30°C to 700°C (heating rate of 20°C/min). Strain-stress curves of lignocellulose bioplastic samples were performed by employing a universal tension machine (Shimadzu, INTRTON5565, Japan). The strain rate was 1 mm/min and the sample size was 30 mm × 10 mm × 2 mm. The water contact angles (θ) of lignocellulose bioplastics were carried out at room temperature by using an optical contact angle analyzer (Biolin, T200 Auto 3 Plus, Sweden). Each sample was tested three times and the average value was calculated. The biodegradability of lignocellulose bioplastics samples was monitored by placing them on the surface of natural soil.

## 3 Results and discussion

### 3.1 Analysis of bamboo leaf components

The composition of bamboo leaves is presented in Table 1. Bamboo leaves exhibit a relatively low cellulose content, while their hemicellulose content is comparatively high. It is evident that the cellulose content of bamboo leaves, when compared to various components found in most types of bamboo trunks (e.g., *Bambusa emeiensis* with approximately 45% cellulose content and *Phyllostachys edulis* with around 50% cellulose content), renders them unsuitable for utilization within the pulp and paper industry (Rusch et al., 2023). However, in the preparation of lignocellulosic bioplastics, a higher hemicellulose (polypentose) content is more favorable for facilitating the dissolution of DES, while an increased lignin concentration can serve as an effective binder to consolidate other lignocellulosic constituents.

**TABLE 1:** Component content of bamboo leaves^a^.

Components	Cellulose (%)	Hemicellulose (%)	Total lignin (%)^b^	Ash (%)	Benzene-alcohol extractive (%)
Content	27 ± 1.0	39 ± 2.4	29 ± 1.1	2 ± 0.1	5 ± 0.5

^a^ The data represent the average of three experiments with standard deviation.

^b^ Total lignin = Klason lignin + acid soluble lignin.

The benzoyl alcohol extract of bamboo leaves exhibits a high content due to the presence of abundant active ingredients, including flavonoids, chlorophyll, amino acids, vitamins, and trace elements alongside a minor proportion of waxy substances. These bioactive compounds possess diverse biological activities such as antibacterial and insecticidal properties (Cheng et al., 2023), thereby offering promising prospects for applications in food preservation and pest control. The benzene alcohol extract of bamboo leaves appears as a dark green hue due to the dissolution of chlorophyll in ethanol or other solvents, which undergoes oxidation upon exposure to air. Consequently, when an equal number of moles of oxygen are absorbed by chlorophyll, the resulting oxidized chlorophyll exhibits a blackish-green coloration.

### 3.2 Chemical characterization of bamboo leaf powder and lignocellulosic bioplastics

**FIGURE 2:**
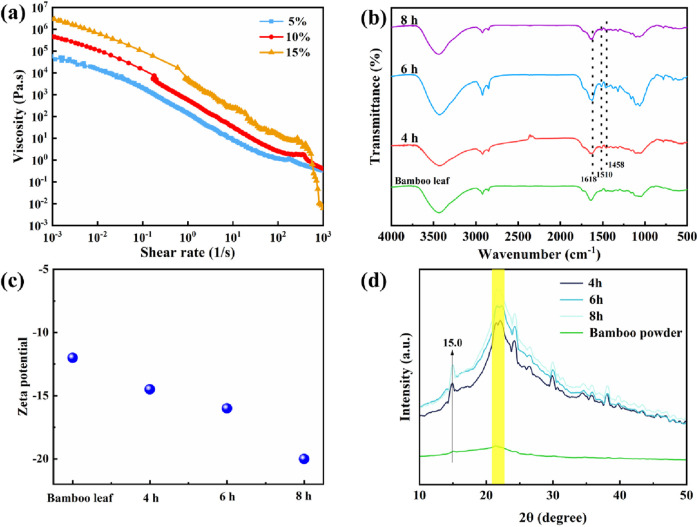
**(A)** Rheological properties of bioplastic treated for varying durations. FT-IR spectra **(B)** and Zeta potential **(C)** of the bamboo leaves and bioplastic treated for varying durations. **(D)** XRD spectra of the bamboo leaves and bioplastic treated for varying durations.

Firstly, a cellulose-lignin slurry was obtained through the regeneration of lignin, and Figure 2A illustrates the viscosity of the slurry with varying solid contents after undergoing washing and filtration processes. The slurry demonstrates a high solid content (15 wt%) along with elevated viscosity. The FT-IR spectra of bamboo leaf powder and bioplastics were examined, as depicted in Figure 2B. The characteristic absorption peaks at 1,458, 1,510, and 1,618 cm^-1^ observed in the lignocellulosic bioplastic indicated the presence of an aromatic skeleton derived from lignin (Ci et al., 2024b). Notably, the intensity of the C = O stretching peak at 1725 cm^-1^ was significantly higher compared to that of bamboo leaf powders, suggesting partial esterification of cellulose hydroxyl groups by oxalic acid during DES treatment. Overall, dissolution and regeneration processes did not induce substantial changes in the chemical structure of lignocellulosic bioplastic. Additionally, the incorporation of carbonyl groups onto cellulose led to bioplastics with varying treatment times of DES, resulting in a more pronounced negative charge compared to bamboo leaf powders (Figure 2C).

The XRD patterns of bamboo leaf and lignocellulosic bioplastics, obtained by subjecting them to different durations of treatment in DES, are presented in Figure 2D. The positions and shapes of the XRD peaks for various bioplastics exhibit a high degree of similarity. Notably, a distinct diffraction peak at 2θ = 15.0° corresponds to the characteristic cellulose type I crystal plane, while another characteristic diffraction peak appears around 21°, indicating that DES treatment induces alterations in the cellulose crystal structure (Liu et al., 2021). The dissolution of lignocellulose by DES is achieved through the cleavage and reformation of glycosidic bonds, facilitated by the establishment of hydrogen bonding interactions between anions, cations, and cellulose (Mamilla et al., 2019). Recrystallization leads to a partial conversion of the cellulose chain into a more thermodynamically stable type II structure, as evidenced by distinct spectral peak patterns that indicate increased crystallinity in the prepared bioplastics. This enhanced crystallinity signifies an improved internal material structure, thereby laying a solid foundation for its exceptional mechanical properties.

### 3.3 Morphology analysis by SEM

**FIGURE 3:**
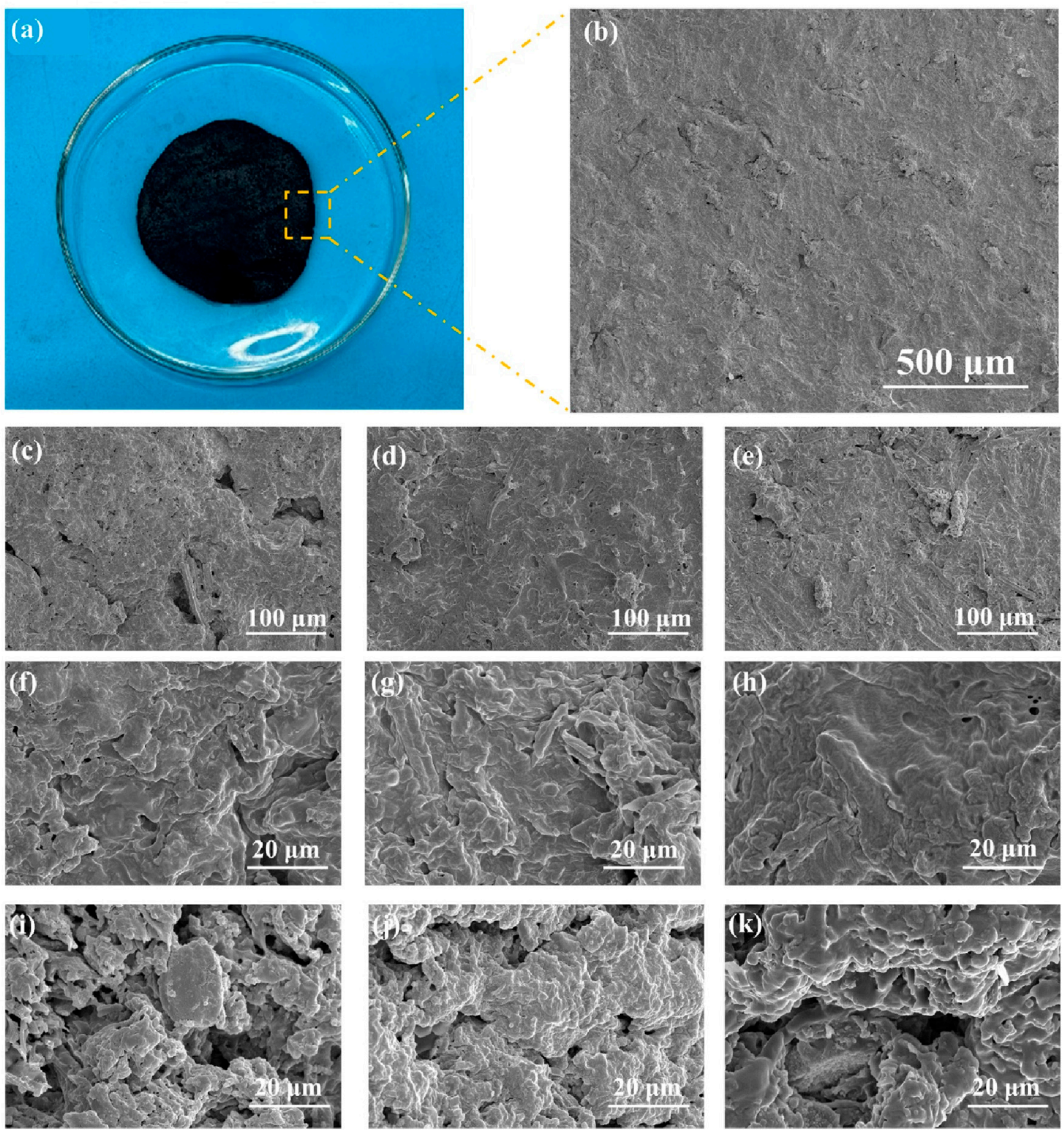
**(A)** Macro view of bioplastics. **(B)** SEM image of the surface of bioplastic. **(C–E)** Surface topography of bioplastics after treatment with DES for 4 h, 6 h, and 8 h at low multiples. **(F–H)** Surface topography of bioplastics after treatment with DES for 4 h, 6 h, and 8 h at higher multiples. **(I–K)** Cross-sectional SEM images of bioplastics after treatment with DES for 4 h, 6 h, and 8 h.

The surface structural characteristics of lignocellulosic bioplastics were investigated by employing SEM to examine the microstructure following various durations of DES treatment. Optical photographs and SEM images (Figures 3A, B) reveal that the lignocellulosic bioplastics exhibit a homogeneous and compact structure, characterized by a relatively smooth surface. The pore size within the bioplastic gradually decreased and the structure became denser as the DES treatment time was extended (4–8 h), as depicted in Figures 3C–E, indicating a significant impact of treatment time on the internal structure density of the plastic. Moreover, Figures 3F–H reveal that the initial cellulose material underwent defibrillation resulting in micro/nano fibrils, which were subsequently coated with regenerated lignin and tightly bonded together. In this process, the incorporation of regenerated lignin, a naturally biodegradable adhesive, effectively enhances fiber-fiber interactions, thereby significantly improving the mechanical properties of the bioplastic. Simultaneously, the regenerated lignin coating exhibited a compact layered structure, thereby endowing the bioplastic with remarkable potential for superior water and oxygen barrier properties as well as efficient water retention capabilities. Figures 3I–K illustrate the cross-sectional morphology of the bioplastic subjected to different DES treatment durations, revealing an intricate network of interlinked structures within the bioplastic matrix. Bioplastics prepared with varying treatment times displayed discernible variations in pore distribution and size, presenting a novel avenue for tailoring bioplastics with adjustable pore architectures.

### 3.4 Analysis of chemical structure of bioplastics

**FIGURE 4:**
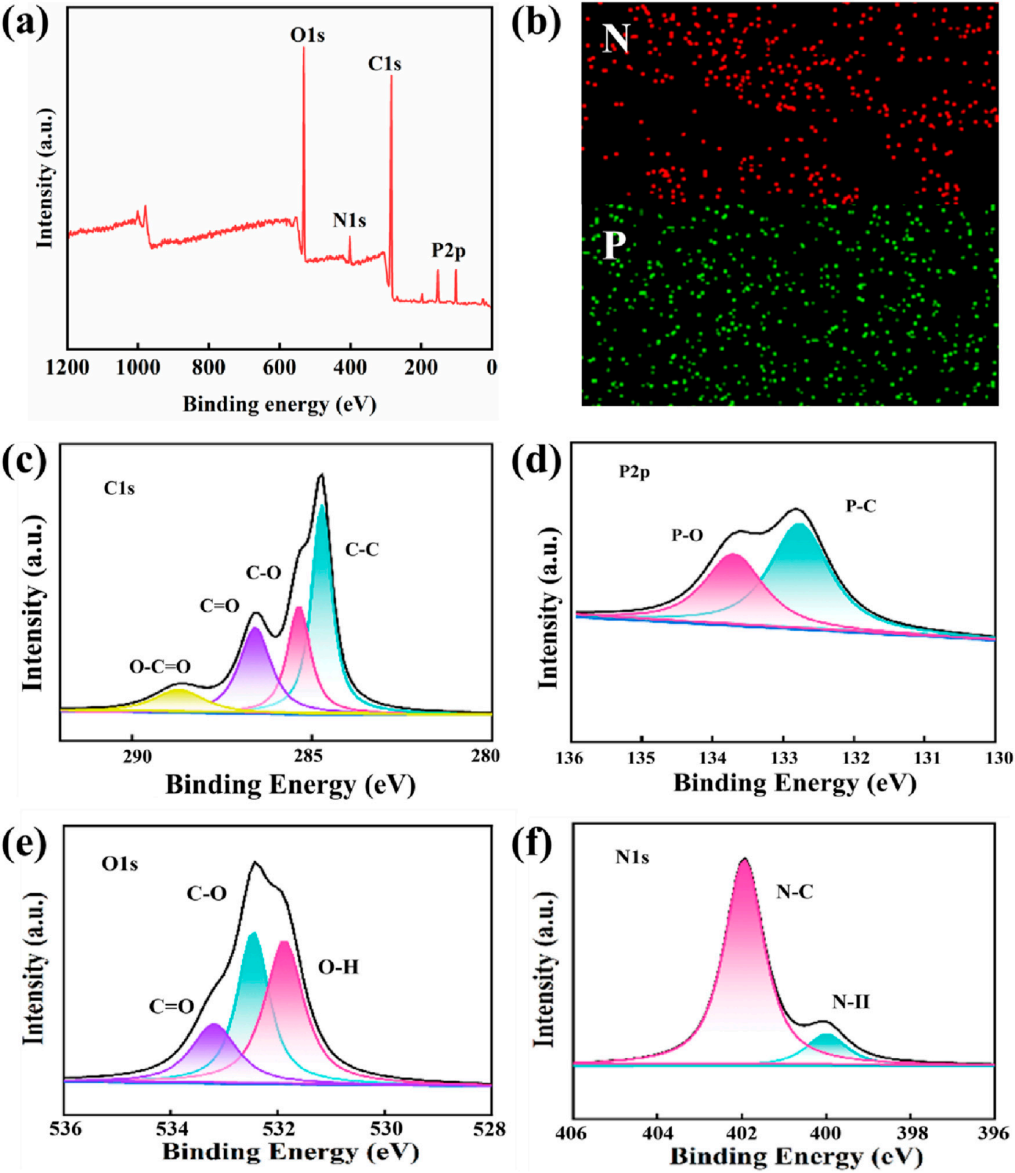
**(A)** XPS wide scan full spectrum. **(B)** N, P EDX mapping of lignocellulosic bioplastics. XPS spectra of lignocellulosic bioplastics. **(C)** C1s peak. **(D)** P2p peak. **(E)** N1s peak. **(F)** O1s peak.

To investigate the potential chemical changes occurring during the preparation of lignocellulosic bioplastics, the samples were analyzed using XPS. Figure 4 shows the full spectrum scan of lignocellulosic bioplastics and the high-resolution deconvolution spectra of key elements. In the wide-scan XPS spectrum (Figure 4A), the characteristic peaks at 532.8, 401.2, 287.5, and 131.5 eV correspond to O 1s, N 1s, C 1s, and P 2p, respectively, confirming the presence of oxygen, nitrogen, carbon, and phosphorus in the bioplastics. Additionally, SEM mapping was employed to further analyze the elemental composition of lignocellulosic bioplastics (Figure 4B). The nitrogen and phosphorus content of lignocellulosic bioplastics were determined to be 1.89% and 0.5%, respectively. The deconvoluted peaks of C 1s further reveal the presence of four carbon chemical environments: C-C (284.8 eV), C-O (285.2 eV), C = O (287.1 eV), and O-C = O (288.5 eV) (Figure 4C). The deconvoluted peaks of P 2p show two phosphorus environments: P-O (133.6 eV) and P-C (132.8 eV) (Figure 4D). The deconvoluted peaks of O 1s suggest that oxygen exists in multiple chemical environments, including C = O (533.2 eV), C-O (532.8 eV), and O-H (531.6 eV) (Figure 4E). The deconvoluted peaks of N 1s indicate the presence of two nitrogen environments in the sample: N-C (401.8 eV) and N-H (400.1 eV) (Figure 4F). These results suggest that functional groups such as carboxyl (-COOH), hydroxyl (-OH), and amino (-NH₂) may be present in the lignocellulosic bioplastics.

### 3.5 Thermal stability and mechanical properties of bioplastics

**FIGURE 5:**
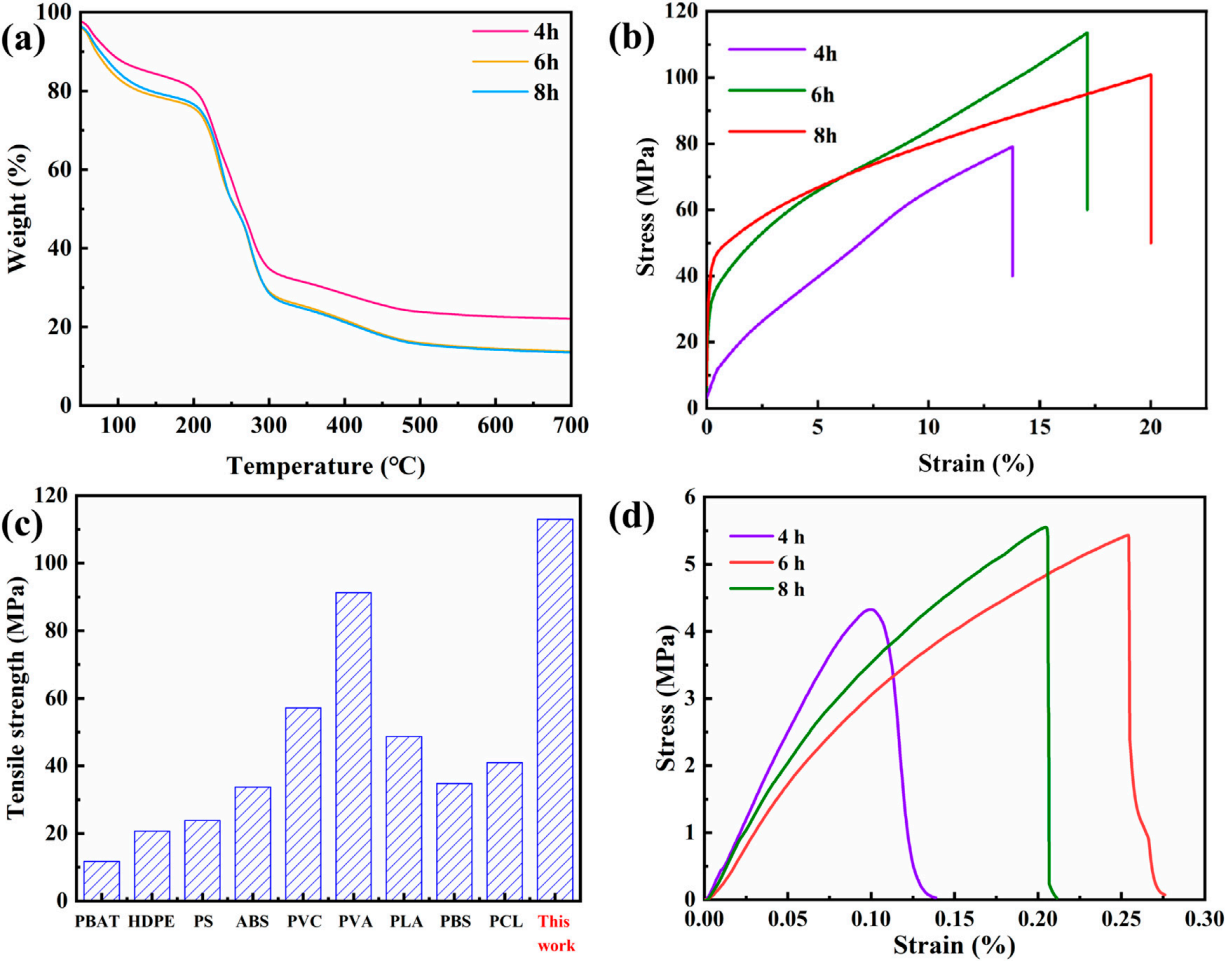
**(A)** Thermal gravimetric analysis images of lignocellulosic bioplastics. **(B)** Variation of dry mechanical properties of the lignocellulosic bioplastics disposed in DES with different times. **(C)** The tensile strength of lignocellulosic bioplastics compared with that of other plastics, including butylene adipate-co-terephthalate (PBAT), high-density polyethylene (HDPE), polystyrene (PS), acrylonitrile butadiene styrene (ABS), poly vinyl chloride (PVC), polyvinyl alcohol (PVA), polybutylene succinate (PBS), and polycaprolactone (PCL) (Xia et al., 2021). **(D)** Variation of wet mechanical properties of the lignocellulosic bioplastics disposed in DES with different times.

Figure 5A shows the TGA curves of lignocellulosic bioplastics. The thermal decomposition process can be divided into four stages. In the first stage (33°C–60°C), the mass loss of the bioplastics is primarily attributed to the gradual evaporation of physically adsorbed water, with minimal mass loss. The mass loss in the second stage (60°C–200°C) may be related to the degradation of a small amount of lignin and the loss of some bound water. When the temperature exceeds 200°C, the process enters the third stage (200°C–300°C), where mass loss increases significantly, reaching the maximum decomposition rate at 296°C. This is mainly due to the carbonization and decomposition of cellulose and lignin. In the fourth stage (300°C–700°C), the remaining cellulose and lignin continue to undergo aromatization and carbonization, with a residue rate of approximately 15%–20%. Additionally, with increasing DES treatment time, the residue rate decreases due to enhanced lignin dissolution and a looser lignocellulosic structure, leading to accelerated pyrolysis of regenerated lignin.

Dry tensile strength and elongation at break are critical indicators for assessing the mechanical properties of bioplastics, essential for ensuring that bioplastics maintain structural integrity and strength in various harsh environments. To this end, the mechanical properties of bioplastics subjected to different treatment times were tested (Figure 5B). The results showed that with extended treatment time, the elongation at break of the bioplastics increased from 13.8% to 20.1%. Meanwhile, the bioplastics subjected to a 6 h treatment exhibited the highest dry tensile strength, reaching an impressive value of 113 MPa, surpassing that of several other bioplastic and petroleum-based counterparts (Figure 5C). This enhancement may be attributed to the entanglement of micro/nanofibers and the adhesive effect of lignin, which collectively improve the mechanical properties of lignocellulosic bioplastics. Lignin, as a natural adhesive, aids in the bonding between fibers, thereby enhancing the overall strength and toughness of the material. These results indicate that optimizing treatment time can effectively improve the mechanical properties of lignocellulosic bioplastics, making them more competitive in practical applications. Additionally, lignocellulosic bioplastics also exhibited satisfactory wet tensile strength (Figure 5D). The bioplastics treated with DES for a duration of 6 h demonstrated a wet tensile strength of 5.4 MPa.

### 3.6 Water contact angle of bioplastics

**FIGURE 6:**
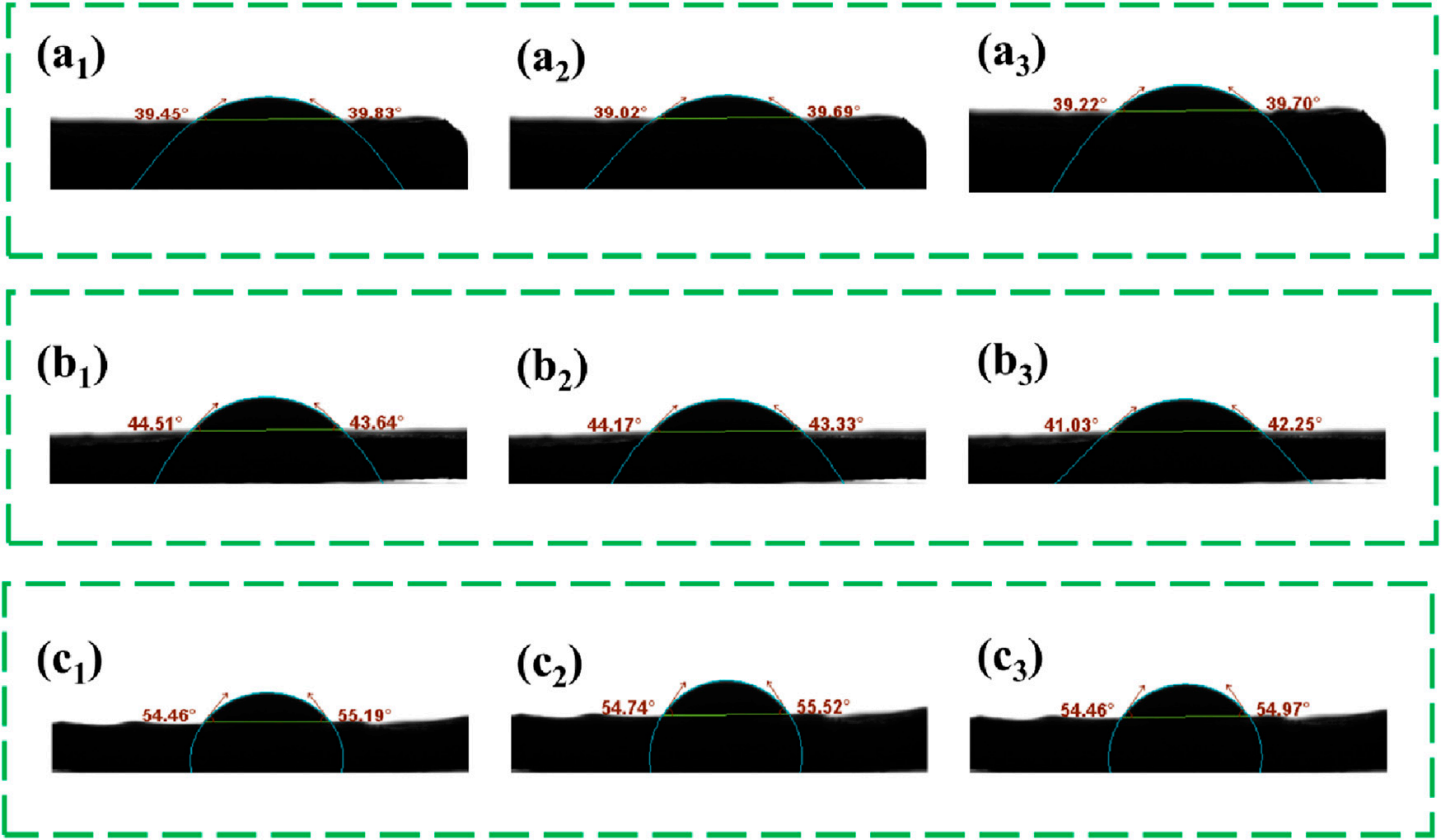
**(A**_**1**_ **–A**_**3**_ **)** The WCA on the surface of bioplastics with DES treated for 4 h **(B**_**1**_ **–B**_**3**_ **)** The WCA on the surface of bioplastics with DES treated for 6 h **(C**_**1**_ **–C**_**3**_ **)** The WCA on the surface of bioplastics with DES treated for 8 h.

As shown in Figure 6, with the extension of treatment time of bamboo leaf powder in DES, the water contact angle of the resulting bioplastic increased from 39.5° (4 h) to 55.52° (8 h). Prolonging the treatment time of the raw material in DES helps to enhance the hydrophobicity of the bioplastic. However, despite the increase in contact angle with extended treatment time, it remained below 90°, indicating hydrophilicity. This phenomenon can be attributed to the increased dissolution of lignin in bamboo leaf powder with longer treatment time. The hydrophobic nature of the phenylpropane units and their carbon chains in lignin facilitates rapid regeneration upon addition of deionized water, enabling integration into the lignocellulosic slurry and thereby augmenting the resulting bioplastic’s hydrophobic properties. Regenerated lignin contains both polar hydrophilic side chains (such as phenolic hydroxyl groups) and non-polar hydrophobic main chains (such as hydrocarbon and phenylpropane). This amphiphilic structure not only imparts unique physicochemical properties to the regenerated lignin but also demonstrates significant advantages in practical applications.

### 3.7 Biodegradability of bioplastics

**FIGURE 7:**
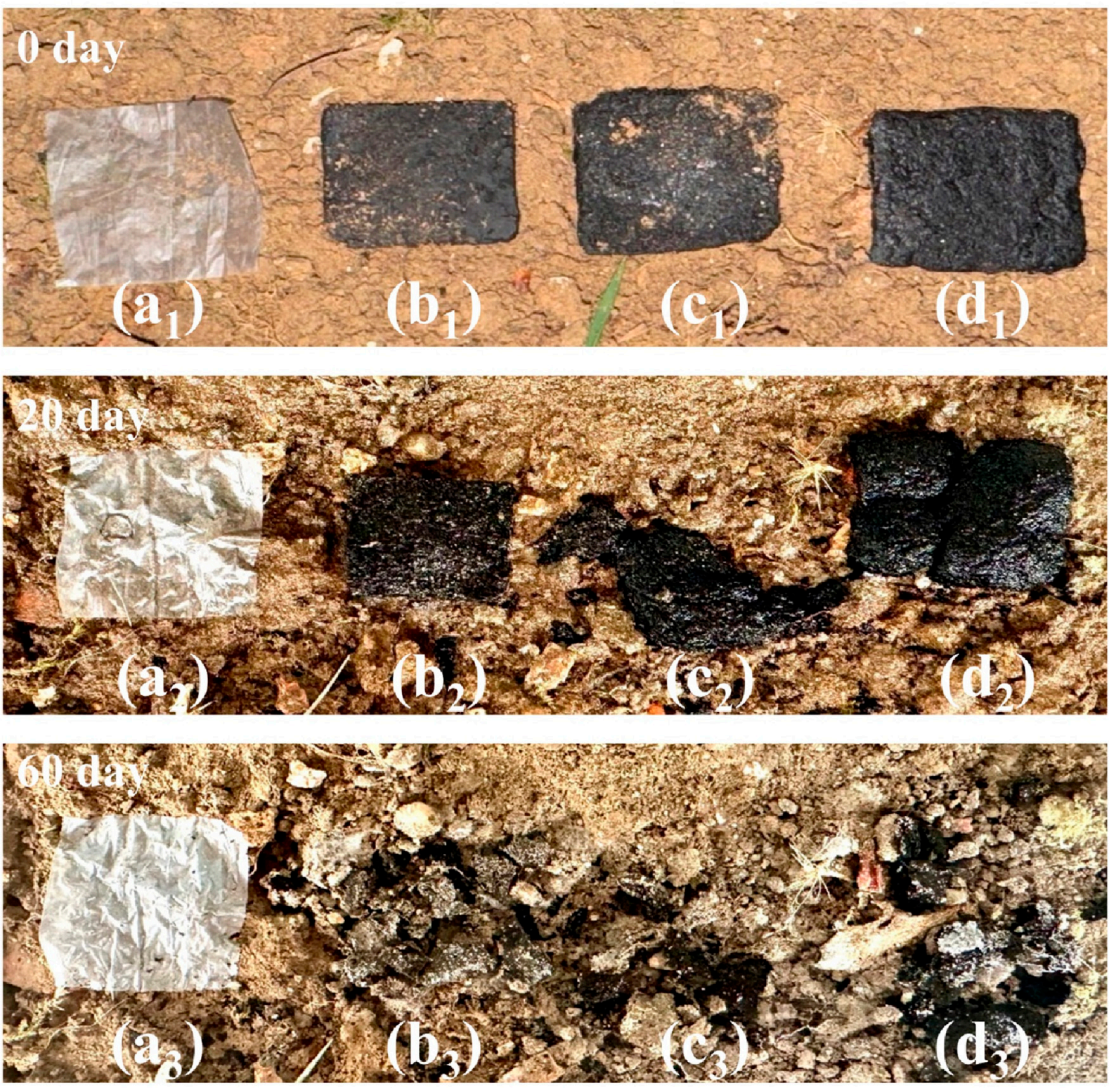
Degradation experiment of bioplastics and petroleum-based plastics. **(A)** Petroleum-based plastics. **(B–D)** Lignocellulose bioplastics with DES treating for 4h–8 h.

The biodegradability of lignocellulose-based bioplastics is crucial for their environmental sustainability and practical applications. To evaluate the prepared bioplastics degradation performance, the biodegradation process of bioplastics in a real soil environment was investigated (as shown in Figure 7). Compared to commercial polyethylene plastics, bioplastics treated with different DES exhibited varying degrees of loosening and fracture after 20 days of exposure in a natural environment, whereas the commercial polyethylene plastics showed no significant changes. The degradation rate of different bioplastics samples exceeded 20% after 20 days. Notably, by the 60th day, these bioplastics had undergone nearly complete degradation with a degradation rate exceeding 98%, thereby further substantiating their exceptional biodegradability. The degradation of bioplastics can be ascribed to three interconnected processes: firstly, the fragmentation caused by soil-dwelling organisms; secondly, the mineralization and humification decomposition facilitated by microorganisms; and finally, the leaching of water-soluble compounds and enzymes. This degradation mechanism reflects the good balance between stability and biodegradability in lignocellulose-based bioplastics. This balance is of significant importance for designing the next-generation of sustainable, biodegradable high-performance plastics. Additionally, lignocellulose-based bioplastics also demonstrate excellent recyclability. After reaching the end of their service life, these bioplastics can be efficiently broken down into a uniform cellulose-lignin slurry through mechanical agitation. This method is not only efficient but also enables lignocellulose-based bioplastics to be recycled as a renewable resource, thereby achieving sustainable resource management.

### 3.8 Possible mechanism

**FIGURE 8:**
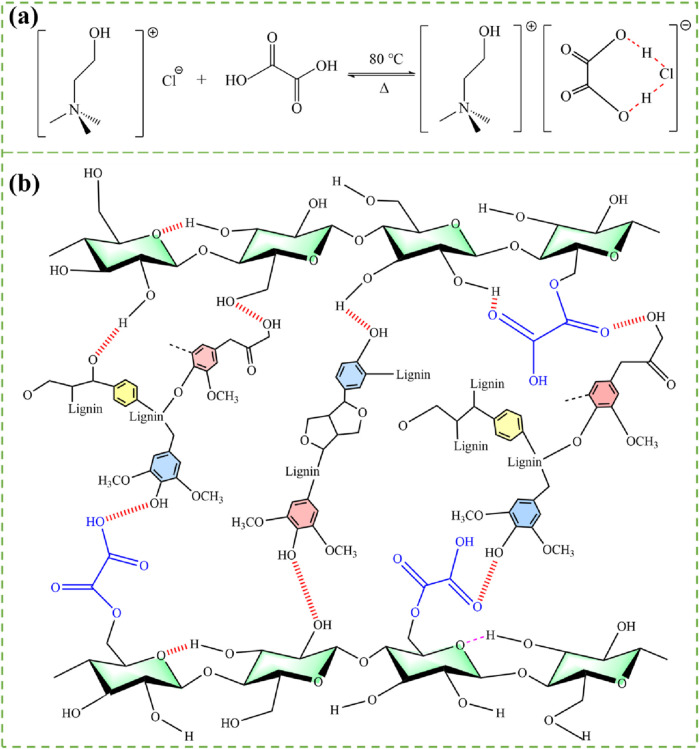
**(A)** The formation of hydrogen bonding between ChCl and oxalic acid. **(B)** The structural linkages between regenerated lignin and cellulose.

As depicted in Figure 8A, the formation of hydrogen bond interactions (OH … Cl) between choline chloride and oxalic acid reduces the crystallization capacity of the compound, thereby maintaining DES stability in its liquid state. Moreover, this structure facilitates the delocalization of hydrogen protons in oxalic acid, leading to an increase in DES acidity and improvement in bamboo leaf powder treatment efficiency (Yu et al., 2022). The strong hydrogen bonding and high acidity of DES facilitate the rapid dissolution of lignin and the dissociation of wood fibers. Subsequently, deionized water, serving as the anti-solvent, was introduced into the DES-lignocellulose dissolution system to facilitate the rapid regeneration of lignin due to its inherent hydrophobicity. The resulting mixture underwent filtration and subsequent washing with water to eliminate any residual DES, yielding a substantial quantity of stable cellulose-lignin slurry. These slurries exhibit elevated solid content and viscosity levels, enabling their utilization in diverse applications such as drying or melting molding processes for the production of bio-based plastics with exceptional properties. In the process of lignin dissolution and regeneration, the aryl ether bonds within lignin undergo cleavage, leading to the formation of novel Hibbert ketones and phenolic hydroxyl groups (Hladnik et al., 2021; Liu Q. et al., 2019). These newly formed ketone and phenol moieties are incorporated into regenerated lignin, facilitating hydrogen bond interactions that promote cross-linking between lignin and cellulose micro/nano fibrils (Figure 8B) (Xia et al., 2021). Consequently, a densely interconnected network structure of lignocellulosic bioplastics is achieved.

## 4 Conclusion

In summary, the biobased plastic was prepared by pretreating bamboo leaf powder with DES and subsequently regenerating lignin *in situ*. Increasing the treatment time of bamboo powder in DES resulted in enhanced crystallinity and improved ordering of the internal structure of the bioplastics. Additionally, a gradual reduction in internal pore size led to a denser structure. When the treatment duration was extended to 6 h, the bioplastic exhibited its highest mechanical strength, with a tensile strength reaching 113 MPa. Moreover, prolonging the treatment time of raw materials in DES resulted in an increased lignin density on the material surface, thereby enhancing water stability of lignocellulosic bioplastics and achieving a maximum contact angle of 55.52°. Additionally, these prepared lignocellulosic bioplastics exhibited remarkable degradability, with a degradation rate surpassing 98% after a duration of 60 days. The present study offers a novel perspective on the production of stable, robust, and biodegradable bioplastics derived from abundant, renewable, and sustainable biomass resources. Furthermore, it highlights the high value utilization of lignin in this process.

## Data Availability

The original contributions presented in the study are included in the article/supplementary material, further inquiries can be directed to the corresponding authors.

## References

[B1] Aziz T. Farid A. Haq F. Kiran M. Ullah A. Zhang K. (2022). A review on the modification of cellulose and its applications. Polymers 14 (15), 3206. 10.3390/polym14153206 35956720 PMC9371096

[B2] Budtova T. Navard P. (2016). Cellulose in NaOH–water based solvents: a review. Cellulose 23 (1), 5–55. 10.1007/s10570-015-0779-8

[B3] Cerro D. R. Koso T. V. Kakko T. King A. W. Kilpeläinen I. (2020). Crystallinity reduction and enhancement in the chemical reactivity of cellulose by non-dissolving pre-treatment with tetrabutylphosphonium acetate. Cellulose 27, 5545–5562. 10.1007/s10570-020-03044-6

[B4] Chen Y.-L. Zhang X. You T.-T. Xu F. (2019). Deep eutectic solvents (DESs) for cellulose dissolution: a mini-review. Cellulose 26, 205–213. 10.1007/s10570-018-2130-7

[B5] Cheng Y. Wan S. Yao L. Lin D. Wu T. Chen Y. (2023). Bamboo leaf: a review of traditional medicinal property, phytochemistry, pharmacology, and purification technology. J. Ethnopharmacol. 306, 116166. 10.1016/j.jep.2023.116166 36649850

[B6] Ci Y. Lv D. Yang X. Du H. Tang Y. (2024a). High-performance cellulose/thermoplastic polyurethane composites enabled by interaction-modulated cellulose regeneration. Carbohydr. Polym. 122611. 10.1016/j.carbpol.2024.122611 39245493

[B7] Ci Y. Ma Y. Chen T. Li F. Tang Y. (2024b). Facile dissolution of cellulose by superbase-derived ionic liquid using organic solvents as co-solvents at mild temperatures. Carbohydr. Polym. 330, 121836. 10.1016/j.carbpol.2024.121836 38368113

[B8] Cole M. Lindeque P. Fileman E. Halsband C. Goodhead R. Moger J. (2013). Microplastic ingestion by zooplankton. Environ. Sci. and Technol. 47 (12), 6646–6655. 10.1021/es400663f 23692270

[B9] Cui J.-Y. Zhang N. Jiang J.-C. (2022). Effects of microwave-assisted liquid hot water pretreatment on chemical composition and structure of moso bamboo. Front. Bioeng. Biotechnol. 9, 821982. 10.3389/fbioe.2021.821982 35198552 PMC8859409

[B10] Dong D. Lei L. Hou Y. Liu C. (2023). Effect of balloon-like structure on properties of bamboo pulp during beating process. Cellulose 30 (12), 7965–7980. 10.1007/s10570-023-05409-z

[B11] Dong T. Chen W. Cai C. Bai F. Zhou Z. Wang J. (2023). Water-stable, strong, biodegradable lignocellulose straws replacement for plastic straws. Chem. Eng. J. 451, 138970. 10.1016/j.cej.2022.138970

[B12] Edgar K. J. Buchanan C. M. Debenham J. S. Rundquist P. A. Seiler B. D. Shelton M. C. (2001). Advances in cellulose ester performance and application. Prog. Polym. Sci. 26 (9), 1605–1688. 10.1016/s0079-6700(01)00027-2

[B13] Hahladakis J. N. Velis C. A. Weber R. Iacovidou E. Purnell P. (2018). An overview of chemical additives present in plastics: migration, release, fate and environmental impact during their use, disposal and recycling. J. Hazard. Mater. 344, 179–199. 10.1016/j.jhazmat.2017.10.014 29035713

[B14] Hladnik L. Vicente F. A. Novak U. Grilc M. Likozar B. (2021). Solubility assessment of lignin monomeric compounds and organosolv lignin in deep eutectic solvents using *in situ* Fourier-transform infrared spectroscopy. Industrial Crops Prod. 164, 113359. 10.1016/j.indcrop.2021.113359

[B15] Khan B. Bilal Khan Niazi M. Samin G. Jahan Z. (2017). Thermoplastic starch: a possible biodegradable food packaging material—a review. J. Food Process Eng. 40 (3), e12447. 10.1111/jfpe.12447

[B16] Koelmans A. A. Redondo-Hasselerharm P. E. Nor N. H. M. de Ruijter V. N. Mintenig S. M. Kooi M. (2022). Risk assessment of microplastic particles. Nat. Rev. Mater. 7 (2), 138–152. 10.1038/s41578-021-00411-y

[B17] Lambert S. Wagner M. (2017). Environmental performance of bio-based and biodegradable plastics: the road ahead. Chem. Soc. Rev. 46 (22), 6855–6871. 10.1039/c7cs00149e 28932844

[B18] Li L. Luo Y. Li R. Zhou Q. Peijnenburg W. J. Yin N. (2020). Effective uptake of submicrometre plastics by crop plants via a crack-entry mode. Nat. Sustain. 3 (11), 929–937. 10.1038/s41893-020-0567-9

[B19] Li Z. Chen C. Mi R. Gan W. Dai J. Jiao M. (2020). A strong, tough, and scalable structural material from fast‐growing bamboo. Adv. Mater. 32 (10), 1906308. 10.1002/adma.201906308 31999009

[B20] Liang W. Liu X. Zheng J. Zhao W. Su C. Ge X. (2022). Insight into crosslinked chitosan/soy protein isolate/PVA plastics by revealing its structure, physicochemical properties, and biodegradability. Industrial Crops Prod. 187, 115548. 10.1016/j.indcrop.2022.115548

[B21] Lindman B. Karlström G. Stigsson L. (2010). On the mechanism of dissolution of cellulose. J. Mol. Liq. 156 (1), 76–81. 10.1016/j.molliq.2010.04.016

[B22] Liu C. Li Y. Gai X. Xiang Z. Jiang W. He S. (2023). Advances in green materials derived from wood for detecting and removing mercury ions in water. Environ. Pollut. 335, 122351. 10.1016/j.envpol.2023.122351 37567404

[B23] Liu C. Li Y. Hou Y. (2019). Effects of alkalinity of ionic liquids on the structure of biomass in pretreatment process. Wood Sci. Technol. 53, 177–189. 10.1007/s00226-018-1066-2

[B24] Liu C. Luan P. Li Q. Cheng Z. Sun X. Cao D. (2020). Biodegradable, hygienic, and compostable tableware from hybrid sugarcane and bamboo fibers as plastic alternative. Matter 3 (6), 2066–2079. 10.1016/j.matt.2020.10.004

[B25] Liu Q. Zhao X. Yu D. Yu H. Zhang Y. Xue Z. (2019). Novel deep eutectic solvents with different functional groups towards highly efficient dissolution of lignin. Green Chem. 21 (19), 5291–5297. 10.1039/c9gc02306b

[B26] Liu Y. Deak N. Wang Z. Yu H. Hameleers L. Jurak E. (2021). Tunable and functional deep eutectic solvents for lignocellulose valorization. Nat. Commun. 12 (1), 5424. 10.1038/s41467-021-25117-1 34521828 PMC8440657

[B27] Luan P. Li J. He S. Kuang Y. Mo L. Song T. (2019). Investigation of deposit problem during sugarcane bagasse pulp molded tableware production. J. Clean. Prod. 237, 117856. 10.1016/j.jclepro.2019.117856

[B28] Mamilla J. L. Novak U. Grilc M. Likozar B. (2019). Natural deep eutectic solvents (DES) for fractionation of waste lignocellulosic biomass and its cascade conversion to value-added bio-based chemicals. Biomass bioenergy 120, 417–425. 10.1016/j.biombioe.2018.12.002

[B29] Nelson M. L. O'Connor R. T. (1964). Relation of certain infrared bands to cellulose crystallinity and crystal lattice type. Part II. A new infrared ratio for estimation of crystallinity in celluloses I and II. J. Appl. Polym. Sci. 8 (3), 1325–1341. 10.1002/app.1964.070080323

[B30] Peelman N. Ragaert P. De Meulenaer B. Adons D. Peeters R. Cardon L. (2013). Application of bioplastics for food packaging. Trends Food Sci. and Technol. 32 (2), 128–141. 10.1016/j.tifs.2013.06.003

[B31] Qin L. Liu Z. Liu T. Liu S. Zhang J. Wu J. (2022). A bioinspired, strong, all-natural, superhydrophobic cellulose-based straw. Int. J. Biol. Macromol. 220, 910–919. 10.1016/j.ijbiomac.2022.08.118 35998858

[B32] Rusch F. Wastowski A. D. de Lira T. S. Moreira K. C. C. S. R. de Moraes Lúcio D. (2023). Description of the component properties of species of bamboo: a review. Biomass Convers. Biorefinery 13 (3), 2487–2495. 10.1007/s13399-021-01359-3

[B33] Schilling M. Bouchard M. Khanjian H. Learner T. Phenix A. Rivenc R. (2010). Application of chemical and thermal analysis methods for studying cellulose ester plastics. Accounts Chem. Res. 43 (6), 888–896. 10.1021/ar1000132 20455567

[B34] Tu H. Zhu M. Duan B. Zhang L. (2021). Recent progress in high‐strength and robust regenerated cellulose materials. Adv. Mater. 33 (28), 2000682. 10.1002/adma.202000682 32686231

[B35] Wu C. Li J. Zhang Y. q. Li X. Wang S. y. Li D. q. (2023). Cellulose dissolution, modification, and the derived hydrogel: a review. ChemSusChem 16 (21), e202300518. 10.1002/cssc.202300518 37501498

[B36] Wu D. Chen J. Lu B. Xiong L. He Y. Zhang Y. (2012). Application of near infrared spectroscopy for the rapid determination of antioxidant activity of bamboo leaf extract. Food Chem. 135 (4), 2147–2156. 10.1016/j.foodchem.2012.07.011 22980783

[B37] Wu Y. Li J. Yu L. Wang S. Lv Z. Long H. (2023). Overwintering performance of bamboo leaves, and establishment of mathematical model for the distribution and introduction prediction of bamboos. Front. Plant Sci. 14, 1255033. 10.3389/fpls.2023.1255033 37746014 PMC10515091

[B38] Xia Q. Chen C. Yao Y. Li J. He S. Zhou Y. (2021). A strong, biodegradable and recyclable lignocellulosic bioplastic. Nat. Sustain. 4 (7), 627–635. 10.1038/s41893-021-00702-w

[B39] Yan M. Zhang L. Ma J. Lu H. Zhou X. (2020). Stable suspensions of lignocellulose nanofibrils (LCNFs) dispersed in organic solvents. ACS Sustain. Chem. and Eng. 8 (42), 15989–15997. 10.1021/acssuschemeng.0c06120

[B40] Yu H. Xue Z. Shi R. Zhou F. Mu T. (2022). Lignin dissolution and lignocellulose pretreatment by carboxylic acid based deep eutectic solvents. Industrial Crops Prod. 184, 115049. 10.1016/j.indcrop.2022.115049

[B41] Yuan J. L. Yue J.-J. Gu X.-P. Lin C.-S. (2017). Flowering of woody bamboo in tissue culture systems. Front. plant Sci. 8, 1589. 10.3389/fpls.2017.01589 28959269 PMC5603696

[B42] Zhao X. Ye H. Chen F. Wang G. (2024). Bamboo as a substitute for plastic: research on the application performance and influencing mechanism of bamboo buttons. J. Clean. Prod. 446, 141297. 10.1016/j.jclepro.2024.141297

[B43] Zhao Y. Lou Z. Wang Q. Yuan T. Chen M. Han H. (2023). Fabrication of a bamboo-based glulam based on reconstitution unit innovation: mechanical property investigation and carbon footprint evaluation. Industrial Crops Prod. 202, 117046. 10.1016/j.indcrop.2023.117046

[B44] Zhu S. Wu Y. Chen Q. Yu Z. Wang C. Jin S. (2006). Dissolution of cellulose with ionic liquids and its application: a mini-review. Green Chem. 8 (4), 325–327. 10.1039/b601395c

[B45] Zimmermann L. Dombrowski A. Völker C. Wagner M. (2020). Are bioplastics and plant-based materials safer than conventional plastics? *in vitro* toxicity and chemical composition. Environ. Int. 145, 106066. 10.1016/j.envint.2020.106066 32951901

